# Smartphone addiction risk, technology-related behaviors and attitudes, and psychological well-being during the COVID-19 pandemic

**DOI:** 10.3389/fpsyg.2022.997253

**Published:** 2022-08-16

**Authors:** Alexandrina-Mihaela Popescu, Raluca-Ștefania Balica, Emil Lazăr, Valentin Oprea Bușu, Janina-Elena Vașcu

**Affiliations:** ^1^Department of Teaching Staff Training, University of Craiova, Craiova, Romania; ^2^Department of Education and Communication Sciences, University of Craiova, Craiova, Romania; ^3^Department of Education Sciences, University of Bucharest, Bucharest, Romania

**Keywords:** smartphone, addiction, psychology, COVID-19, pandemic

## Abstract

COVID-19 pandemic-related perceived risk of infection, illness fears, acute stress, emotional anxiety, exhaustion, and fatigue, psychological trauma and depressive symptoms, and sustained psychological distress can cause smartphone addiction risk and lead to technology-related cognitive, emotional, and behavioral disorders, thus impacting psychological well-being. Behavioral addiction of smartphone users can result in anxiety symptom severity, psychiatric symptoms, and depressive stress. We carried out a quantitative literature review of the Web of Science, Scopus, and ProQuest throughout June 2022, with search terms including “smartphone addiction + COVID-19” + “stress,” “anxiety,” “depression,” “psychological distress,” “screen time,” and “fear.” As we analyzed only articles published between 2020 and 2022, 288 papers met the eligibility criteria. By excluding sources with similar titles, having unclear findings or unsupported by replication, or displaying inconsistent content, we selected 64, mainly empirical, sources. We used layout algorithms (VOSviewer) and bibliometric mapping (Dimensions) as data visualization tools. Assessing the Methodological Quality of Systematic Reviews (AMSTAR), a systematic review and literature review software (Distiller SR), Mixed Methods Appraisal Tool (MMAT), and Systematic Review Data Repository (SRDR) were employed as methodological quality assessment tools. As limitations, we analyzed only articles published between 2020 and 2022 in scholarly outlets indexed in the Web of Science, Scopus, and ProQuest databases. The scope of our study also does not advance the inspection of sources covering vulnerable individuals suffering from certain diseases or specific generations. Subsequent analyses should develop on smartphone use and addiction among children and adolescents. Future research should thus investigate problematic smartphone use and addiction across generations Z and Alpha. Attention should be directed to their personality traits and psychopathological symptoms.

## Introduction

The addictive relationship with technological devices (Masaeli and Farhadi, 2021; Serra et al., 2021; Chopdar et al., 2022; Hu et al., 2022; de Freitas et al., 2022a; Zhao et al., 2022a) has been intensified by the consequences of the COVID-19 outbreak. By impacting health and well-being, the COVID-19 crisis has contributed to smartphone addiction. Significant detrimental psychological and behavioral consequences due to the COVID-19 pandemic associated with infection control measures result in spending more time at home and thus increasingly using technological devices (Green, 2020; Wallace and Lăzăroiu, 2021; Lăzăroiu et al., 2021a,b; Blake, 2022) that also configure psychological and social support. Smartphone use disorder is associated with isolation and mental health issues. COVID-19 phobia and news exposure impact problematic smartphone use (PSU) and pessimism, shaping mobile shopping frequency. Smartphone addiction risk is reduced with the COVID-19 pandemic under control.

Excessive social media and PSU during COVID-19 home quarantines (Hallauer et al., 2021; Hu et al., 2021; Köse and Murat, 2021; Saiful et al., 2021; Zhang et al., 2021) are associated with having urban residence, watching television, irregular physical exercise, and poor sleep and psychological well-being. Functional impairment is associated with behavioral addictions such as PSU that mediates the link between COVID-19-related exposure and post-traumatic stress disorder (PTSD) symptoms. Smartphone overuse by constantly *checking* such technological devices (Johnson and Nica, 2021; Platt, 2021; Kliestik et al., 2022b) is associated with increased cyberchondria level. PSU is considerably related to sleep disturbance and physical and mental fatigue, increasing negative health behaviors.

## Methodology

**Figure 1:**
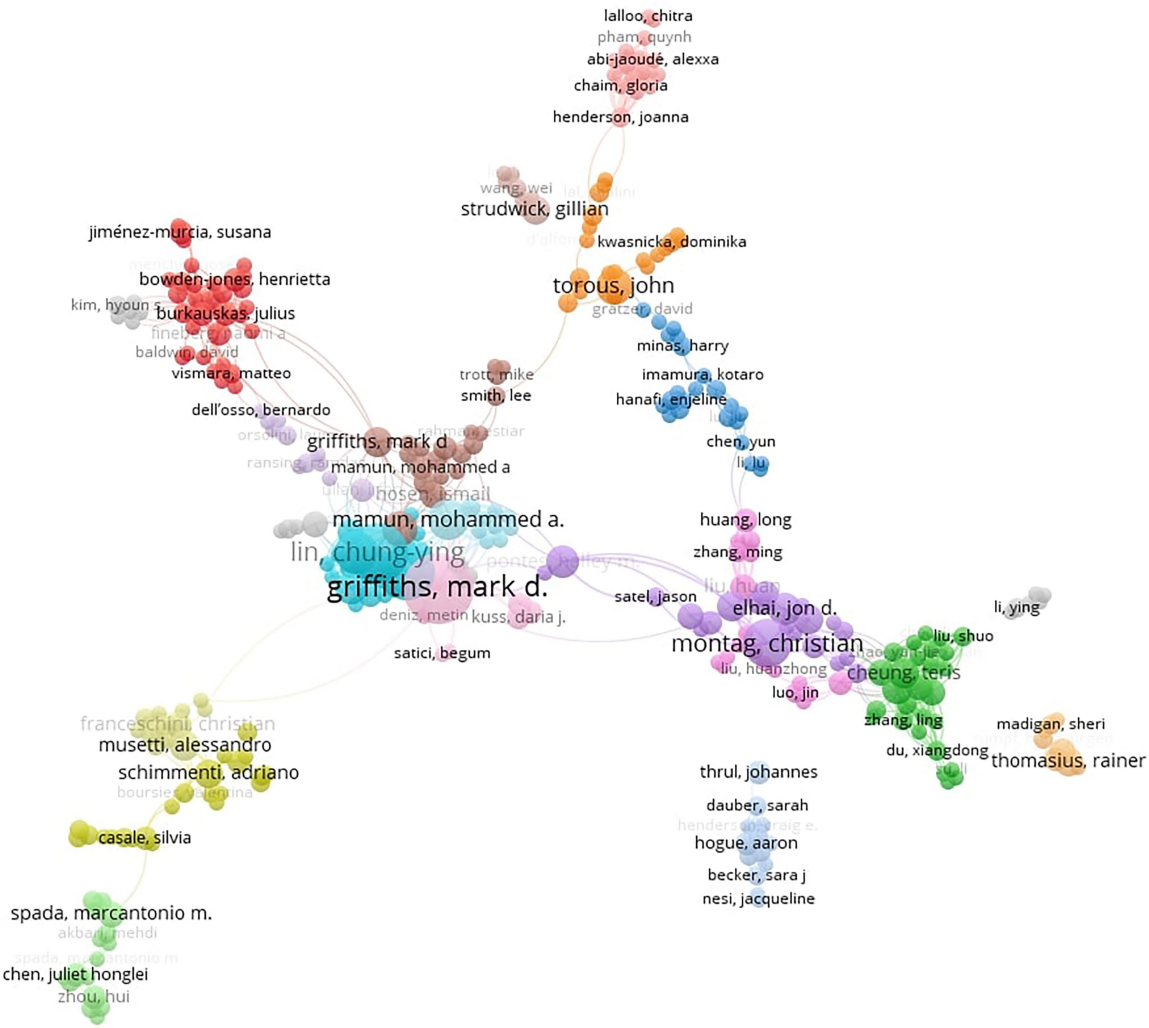
Co-authorship covering the topic.

We carried out a quantitative literature review of the Web of Science, Scopus, and ProQuest databases throughout June 2022, with search terms including “smartphone addiction + COVID-19” + “stress,” “anxiety,” “depression,” “psychological distress,” “screen time,” and “fear,” that is the most employed words/phrases throughout the inspected literature. As we analyzed only articles published between 2020 and 2022, 288 papers met the eligibility criteria. By excluding sources with similar titles, having unclear findings or unsupported by replication, or displaying inconsistent content, we selected 64, mainly empirical, sources (Table 1). We used layout algorithms (VOSviewer) and bibliometric mapping (Dimensions) as data visualization tools (Figure 1; covering co-authorship). Assessing the Methodological Quality of Systematic Reviews (AMSTAR), a systematic review and literature review software (Distiller SR), Mixed Methods Appraisal Tool (MMAT), and Systematic Review Data Repository (SRDR) were employed as methodological quality assessment tools. No institutional ethics approval was needed, as we analyzed only publicly accessible documents.

**Table 1:** Topics and types of scientific products identified and selected.

**Topic**	**Identified**	**Selected**
smartphone addiction + COVID-19 + stress	62	12
smartphone addiction + COVID-19 + anxiety	61	13
smartphone addiction + COVID-19 + depression	55	13
smartphone addiction + COVID-19 + psychological distress	53	12
smartphone addiction + COVID-19 + screen time	29	7
smartphone addiction + COVID-19 + fear	28	7
**Type of paper**		
Original research	198	61
Review	16	3
Conference proceedings	23	0
Book	14	0
Editorial	37	0

Processed by the authors. Some topics overlap.

## Stress, anxiety, and depression associated with smartphone overuse

Internet-based addictive behaviors have intensified throughout the COVID-19 lockdowns (Drouin et al., 2020; Huixi et al., 2020; Liu et al., 2021; Masaeli and Farhadi, 2021; Nan and Guangyu, 2021; Lai et al., 2022; Ma et al., 2022; Peng et al., 2022; de Freitas et al., 2022b), leading to stress, anxiety, and depression. Smartphone addiction is related to stress and to unsatisfactory sleep patterns. Coping style, stress, anxiety, and depression can mediate the link between resilience and smartphone addiction. COVID-19 stress is an essential determinant of addictive social media use—relationship serially mediated by excessive smartphone use and online connectivity. Users who undergo increased COVID-19 stress are at high risk of addictive social media use that can be furthered by active access and flow experience. Frequency and duration of smartphone use are correlated with stress, anxiety, and depression, in addition to female gender and age. Psychological vulnerabilities and detrimental behavioral actions associated with social distancing increase technology use (Balica, 2021; Lăzăroiu and Harrison, 2021; Nemțeanu et al., 2021; Hopkins, 2022) in terms of COVID-19 information seeking and lead to stress and anxiety. Rumination mediates, and self-control moderates, the link between perceived stress and smartphone addiction. Perceived stress is positively related to nomophobia, while social support to some extent mediates the link between perceived stress and nomophobia. Stress can be moderated by social support in persons having increased levels of nomophobia.

Smartphone addiction has been heightened by the COVID-19 crisis, affecting physical and mental health (Elhai et al., 2020; Haddad et al., 2021; Hallauer et al., 2021; Yan, 2021; Zhan et al., 2021; Li et al., 2021a; Güldal et al., 2022; Song et al., 2022; Liu et al., 2022a), and resulting in clinical anxiety symptoms. COVID-19 anxiety is related to PSU severity, e.g., through excessive news exposure, and mediates the link between general anxiety and PSU severity. Anxiety sensitivity mediates links between anxiety and PSU severity. Anxiety generated by COVID-19 home confinement is related to PSU and sleep disturbance. Neuroticism impacts smart addiction by online social anxiety, while extraversion influences PSU through cyber danger belief. Anxiety, depression, smartphone use in bed, and alcohol consumption are associated with smartphone addiction. PSU is associated with poor mental health outcomes (e.g., anxiety and depression), but cognitive reappraisal and optimism can improve psychological well-being. Psychological flexibility is influenced by COVID-19 anxiety, smartphone addiction, and humor coping. Problematic social media usage predicts the level of anxiety.

Problematic and excessive use of technological devices (Birtus and Lăzăroiu, 2021; Campbell and Popescu, 2021; Cohen and Nica, 2021) during COVID-19-related confinement orders has adversely influenced behavioral health (Duan et al., 2020; Hosen et al., 2021; Jahan et al., 2021; Jin et al., 2021; Yang et al., 2021; Karaoglan Yilmaz et al., 2022; Zhang et al., 2022), leading to anxiety, depression, and smartphone addiction associated with severe Internet dependency. Loneliness associated with COVID-19 lockdowns shapes psychological and social issues such as aggression behaviors and PSU. Social loneliness is positively related to depressive symptoms. Boredom and emotional loneliness are positively related to both smartphone addiction and depressive symptoms. COVID-19 quarantine and lockdown are not considerably associated with smartphone addiction or depressive symptoms. Smartphone addiction issues are related to anxiety, depressive, and insomnia symptoms. Performing almost no physical activity and increased social media-based online engagement impact behavioral health and lead to PSU, in addition to anxiety and depression. Smartphone addiction, living in urban areas, emotional distress, clinical depressive symptoms, lockdown and social distancing measures, and having loved ones infected with COVID-19 are related to elevated anxiety levels. Smartphone addiction, separation anxiety levels, prior mental health issues, and emotion-focused coping style are related to depressive symptoms. Depression and anxiety symptoms predict PSU, while perceived social support and resilience constitute protective factors.

## Smartphone addiction and psychological distress

Incidence of COVID-19 infection, public health campaigning, and preventive measures (Kufel, 2020; Brailovskaia et al., 2021; Elhai et al., 2021; Fernandes et al., 2021; Ting and Essau, 2021; Waliszewski and Warchlewska, 2021; Zhen et al., 2021; Li et al., 2021b; Altamih and Elmahi, 2022; Liang et al., 2022; Sarıalioğlu et al., 2022; Zhao et al., 2022b) are associated with psychological distress. Psychological distress and COVID-19 anxiety are related to significant social media use due to home confinement, in addition to addictive behaviors associated with food and online gaming. Low confidence, psychological distress, and loneliness predict PSU, impacting emotional well-being during COVID-19 lockdown restrictions. Socially isolated individuals are likely to be companionless and consequently use their technological devices to fulfill their relationship needs, increasing their smartphone addiction risk. Social isolation has a positive indirect impact on smartphone addiction *via* loneliness. Smartphone addiction increases with the growing level of loneliness. Escape motivation mediates the link between loneliness and PSU, whereas self-control moderates the link between escape motivation and PSU: as self-control intensifies, escape motivation is improbable to induce PSU (loneliness and escape motivation may increase the risk of PSU). PSU severity is negatively related to sense of control and positively associated with fear of missing out (FoMO), repetitive negative thinking (RNT), and daily time spent on technological devices. The adverse link between sense of control and PSU severity is mediated by FoMO. RNT moderates the positive link between FoMO and PSU severity: the more increased the RNT, the more powerful is the relationship between FoMO and PSU. FoMO and boredom proneness mediate the link between psychological distress and PSU, shaping COVID-19-related mental health and behavior. FoMO mediates links between health anxiety and both PSU and gaming disorder severity. The relationship between COVID-19 stressors and PSU is mediated by FoMO. Regulatory emotional self-efficacy moderates the association between FoMO and PSU, and the link between COVID-19 stressors and PSU. COVID-19 stressors are positively related to FoMO and to PSU. FoMO is positively associated with PSU. Regulatory emotional self-efficacy is negatively related to PSU, while decreasing the links between COVID-19 stressors and FoMO on PSU. In individuals having reduced regulatory emotional self-efficacy, FoMO impacts PSU. FoMO has a significant effect on PSU among persons having increased regulatory emotional self-efficacy. The effect of FoMO on PSU is moderated by regulatory emotional self-efficacy. Individuals experiencing an increased level of COVID-19 stressors are likely to engage in PSU. FoMO mediates the link between COVID-19 stressors and PSU. Persons having increased regulatory emotional self-efficacy rarely resort to PSU for psychological satisfaction. The effect of COVID-19 stressors on PSU is more significant for individuals having increased regulatory emotional self-efficacy. If such persons who are under a relevant level of COVID-19 stressors expect too much of their sense of control as regards technological device use or minimize the repercussions of smartphone addiction, regulatory emotional self-efficacy may exacerbate their PSU. With the decrease of negative emotions brought about by COVID-19 stressors, the PSU risk can be diminished.

## Excessive screen time and social media overuse

Behavioral addiction of smartphone users (Saadeh et al., 2021; Serra et al., 2021; Chopdar et al., 2022; Hu et al., 2022; de Freitas et al., 2022b) can result in anxiety symptom severity, psychiatric symptoms, and depressive stress. Smartphone addiction risk can configure pathological personality traits related to stress, anxiety, and depression (e.g., compulsive smartphone usage, depression psychopathology, and social anxiety). COVID-19 lockdown impacts mental health (Montag and Elhai, 2020; Chen et al., 2021; Hodes and Thomas, 2021; Werling et al., 2021; Adachi et al., 2022; Sui et al., 2022) through an escalation in screen media use. As the chief reason of screen time increase has been the growing smartphone use, reduced deployment of digital devices have positive consequences on depressive symptoms. Digital technology overuse through spending immoderate time in front of smartphones constitutes a direct result of the COVID-19 crisis. Excessive screen time is typified by incessant digital use without regard to adverse effects (e.g., psychopathological states). Excessive screen time and social media overuse predicts nomophobia and smartphone addiction during the COVID-19 pandemic. Fear of COVID-19 infection can increase psychological distress and screen time use, resulting in problematic social media and smartphone use. COVID-19 lockdown and social distancing have led to digital technology overuse (Lewis, 2021; Nica, 2021; Pera and Cuțitoi, 2021; Nemțeanu et al., 2022) in terms of screen time, consequently impacting mental health. Prolonged screen time negatively affects mental health during the COVID-19 crisis, possibly leading to depressive symptoms. Smartphone attachment mediates the link between screen time and depression.

## Fear of COVID-19 and smartphone addiction

Fear of COVID-19 leads to psychological and social problems (De Pasquale et al., 2021; Elhai et al., 2021; Kayis et al., 2021; Mihalca et al., 2021; Montag et al., 2021; Yam et al., 2021; Liu et al., 2022a), having both a direct and indirect impact on mental wellbeing. Fear of COVID-19 is related to decreased mental wellbeing by adversely impacting persons’ emotion and behavior, and impacts mental wellbeing *via* loneliness and smartphone addiction. A reduction in social interactions due to quarantine measures affects mental health and wellbeing, while fear of COVID-19 can result in increased loneliness associated with smartphone addiction that adversely impacts quality of life. Prior emotional and behavioral issues are intensified by the COVID-19 pandemic, and fear of such a contagious virus may cause the affected persons to interpret risky situations as a source of apprehension wearisome to cope with, thus increasing self-isolation behavior and feelings of loneliness. Smartphone addiction and loneliness can be associated with COVID-19 misinformation, leading to maladaptive feelings and behaviors, while emotional awareness would diminish stress and anxiety. Psychosocial support in relation to excessive smartphone use would improve mental wellbeing and physical health, while easing loneliness and emotional and behavioral disorders. Consequently, fear of COVID-19 can bring about loneliness that can result in smartphone addiction risk, causing decreased mental wellbeing, mood, and sleep quality. Technological addictions through intensive smartphone use have aggravated fear of COVID-19, in addition to social isolation and seeking out health information, causing cyberchondria behaviors. There is a positive link between fear of COVID-19, smartphone addiction, and cyberchondria severity. Cyberchondria severity has both a moderating and a mediating role in the link between fear of COVID-19 and smartphone addiction. Fear and anxiety in relation to COVID-19 are increased by self-isolation and social distancing measures. Social networks use disorder partially mediates the link between fear of COVID-19 and smartphone use disorder. Neuroticism constitutes a risk factor as regards fear of COVID-19, social networks use disorder, and smartphone use disorder. Fear of COVID-19 contagion has caused perceived vulnerability, insomnia, stress, anxiety, depression, and PSU, while home quarantine and social distancing measures have diminished social contacts and networks, leading to poor interaction and unsatisfactory psychophysical well-being. Subhealth and insomnia are adversely related to smartphone addiction.

## Discussion

COVID-19 pandemic-related perceived risk of infection, illness fears, acute stress, emotional anxiety, exhaustion, and fatigue, psychological trauma and depressive symptoms, and sustained psychological distress can cause smartphone addiction risk (Montag and Elhai, 2020; Chen et al., 2021; Adachi et al., 2022) and lead to technology-related cognitive, emotional, and behavioral disorders, thus impacting psychological well-being. We integrate our review throughout research indicating that intensified negative emotionality is related to more smartphone addiction (Caponnetto et al., 2021; Hu et al., 2021, 2022; Sfeir et al., 2021; Marengo et al., 2022; de Freitas et al., 2022a; Zhao et al., 2022a), leading to addictive behavior development, whereas increased confidence and household crowding are associated with decreased smartphone addiction during the COVID-19 lockdown periods. Smartphone addiction has brought about impaired social interactions and relationships, with COVID-19 lockdown periods leading to pathological use of technological devices (Barnes, 2021; Nemțeanu and Dabija, 2021; Balica et al., 2022; Zvarikova et al., 2022; Nica et al., 2022a) and thus to developing mental issues. Interpersonal alienation predicts smartphone addiction. Meaning in life shapes interpersonal alienation and smartphone addiction. Refusal self-efficacy moderates the impact of boredom proneness on pathological use of technological devices. Adaptive behavior in relation to smartphones would improve mental health during the COVID-19 pandemic. Instant messaging app and social network excessive use (Mullen and Lythberg, 2021; Blazek et al., 2022; Kliestik et al., 2022a; Nica et al., 2022b) may cause behavioral addictions. COVID-19 lockdowns and maintaining social distance have increased psychological and behavioral issues such as daytime sleepiness, PSU, and PTSD.

Technology-related behaviors and attitudes associated with smartphone addiction risk (Lăzăroiu et al., 2020) can lead to social anxiety symptom severity and depression psychopathology. Smartphone use disorder develops on the behavioral dynamics of depression and anxiety symptoms. Our research complements recent analyses clarifying that smartphone addiction has dramatically worsened (Zhang et al., 2021; Zhao et al., 2021, 2022b; Jiang et al., 2022; Meng et al., 2022; Potas et al., 2022; Xu et al., 2022; Zarco-Alpuente et al., 2022) throughout the COVID-19 outbreak. Smartphone overuse can adversely influence physical and mental health. Causing insomnia, decreased life satisfaction, and increased tobacco and alcohol use, smartphone addiction can lead to severe psychological injuries. Low life satisfaction and insomnia can cause PSU. COVID-19 stressors are positively associated with PSU. Increased online engagement may adversely impact mental and physical health for vulnerable individuals during COVID-19 mandatory self-isolation and lockdown measures, resulting in PSU. Extensive stringent isolation policies enable social distancing but may lead to PSU and intensify smartphone addiction risk, shaping mental health, and physical behavior. Sleep quality mediates the link between PSU and daytime fatigue among quarantined individuals.

## Conclusion

Relevant research has investigated whether smartphone usage throughout the COVID-19 home quarantines (Saadeh et al., 2021; Serra et al., 2021; Chopdar et al., 2022; Hu et al., 2022; de Freitas et al., 2022b) has greatly increased. Unfavorable clinical, psychological, and social outcomes generated by smartphone overuse during the COVID-19 pandemic differ according to degrees of addiction. Social influence is pivotal in moderating mobile shopping frequency for persons bearing the brunt of smartphone addiction. Decreased interpersonal alienation levels can enhance meaning in life and decrease smartphone addiction risk throughout public health emergencies. Increased time spent with technological devices has led to problematic lifestyles and less adaptive behavior.

The research outcomes drawn from the analyzed sources indicate that COVID-19-related preventative behaviors (e.g., home quarantine) have intensified affected persons’ reliance on smartphones (Hu et al., 2021; Sfeir et al., 2021; Zhan et al., 2021; Potas et al., 2022; Liu et al., 2022b) by reducing social interactions. Self-esteem mediates the link between negative emotionality and smartphone addiction. Daytime sleepiness mediates the link between COVID-19-related exposure, PSU, and PTSD symptoms. Smartphone use before sleep and unsatisfactory mental health are associated with addiction to technological devices. Assessing awareness, attitudes, and behavior in relation to smartphone addiction is pivotal in users’ physical and psychological well-being.

## Limitations, implications, and further directions of research

As limitations, we analyzed only articles published between 2020 and 2022 in scholarly outlets indexed in the Web of Science, Scopus, and ProQuest databases. The scope of our study also does not advance the inspection of sources covering vulnerable individuals suffering from certain diseases or specific generations. Subsequent analyses should develop on smartphone use and addiction among children and adolescents. Future research should thus investigate PSU and addiction across generations Z and Alpha. Attention should be directed to their personality traits and psychopathological symptoms.

## Author contributions

All authors listed have made a substantial, direct, and intellectual contribution to the work and approved it for publication.

## Conflict of interest

The authors declare that the research was conducted in the absence of any commercial or financial relationships that could be construed as a potential conflict of interest.

## Publisher’s note

All claims expressed in this article are solely those of the authors and do not necessarily represent those of their affiliated organizations, or those of the publisher, the editors and the reviewers. Any product that may be evaluated in this article, or claim that may be made by its manufacturer, is not guaranteed or endorsed by the publisher.
